# Preventive Dentistry

**Published:** 1905-04

**Authors:** C. M. Wright

**Affiliations:** Cincinnati, Ohio


### PREVENTIVE DENTISTRY.[1]

BY C. M. WRIGHT, A.M., D.D.S., CINCINNATI, OHIO.[2]

Preventive Medicine and Preventive Dentistry are terms of comparatively recent origin, but the prevention of disease has been the foundation principle upon which medicine and dentistry have stood since the earliest times.

The crudest efforts of the primitive dentist to stop decay by driving lead into a hole in a tooth, and the clumsiest attempts on his part to replace lost teeth with ivory, or animals' teeth, or porcelain, must be recognized as operations in preventive dentistry.

Miller's magnificent results in etiology, Bryan's experiments with the systematic application of nitrate of silver, D. D. Smith's repeated and frequent polishing of every exposed surface of every tooth, Michel's analyses of the varying salivary and oral secretions of immunes and the other classes, Black and Johnson's extensions of fillings, Jenkins's artistic creations in porcelain, in short, every individual suggestion from every thinking and working man in the profession of dentistry, discloses the central idea which inspires him. It is the prevention of oral or dental disease.

We have been so busy with actual disease, or disease which is apparent, that we have not given adequate attention to *potential disease.* This must engage our earnest attention for the future.

The coming dentist must operate upon sound teeth and prevent the outbreak of diseases which are lying dormant,—the latent diseases which observation has shown will almost surely appear.

The old preventive dentistry, in which we are all so earnestly engaged, is not to be forgotten, but the modern dentist must, by subtile chemical and surgical means, skilfully and scientifically applied upon sound organs, make these operations unnecessary.

In this field—this field called prophylaxis—the weapons of defence are advanced knowledge of physiology, of etiology and pathology, and of a new therapeutics.

When preventive medicine succeeds in reducing the percentage

---

[1] Read at the Union Meeting in Basel, Switzerland, December 18, 1904.

[2] Professor of Physiology and Pathology in the Ohio College of Dental Surgery.

of deaths from typhoid, small-pox, diphtheria, and tuberculosis,—I said percentage,—the physician's heart is filled with hope and joy. He feels the thrill of courage stimulating him to persevere.

The success which has crowned dentistry as a unique profession during its brief existence, or since the establishment of the first dental school, is so great that we have reason for our faith in its future in this new field.

Prophylaxis, or active measures for the preservation of the health and the prevention of disease, presents special opportunities in dentistry. The necessary manipulations can be readily applied. The importance of them can be certainly demonstrated. Governments and the heads of schools can be easily convinced of the public utility of them. Its wide-reaching effects on the general health of citizens can be proved. If properly presented by qualified dentists, oral prophylactic measures can be made so popular that communities will adopt them and be appreciably benefited. Oral prophylaxis is in line with the most advanced ideas of sanitary science and preventive special and general medicine. A meeting like the present, composed of delegates or members of some of the most important Dental Societies of the civilized world, and meeting in this beautiful city on the Rhine, a city noted for her distinguished medical scientists and practitioners, is, it seems to me, the society and place *par excellence* for the inauguration of a new scientific method. Let a practical method be adopted here to-day, and the name of dear old Basel will be forever associated with a reformation in dental practice. I have special personal reasons for my love of this city and her people, as Dr. Bryan, your president, and others know. Therefore, permit me to suggest that a committee of one or two resident dentists of Basel be appointed to take the matter in hand by authority of this society. This committee shall confer with the sanitary officials of the city, and make arrangements for the establishment of a class or classes of children selected from some eleemosynary institution, or from the public schools, and under the inspection or observation of members of the Sanitary Board; the object being to demonstrate that by Dr. Bryan's method of applying the protective solution of nitrate of silver, and Dr. D. D. Smith's method of fortnightly or monthly polishing of the teeth, dental caries can be positively prevented—seventy-five to ninety per cent.

Secondly, that this same method be followed by other committees in other cities in Europe, having delegates now in attendance at this congress.

Medical men recognizing the pathogenetic magnitude of oral sepsis in relation to other contiguous and even remote tissues, will become the most interested spectators of the exposition. This is not an impossible proposition when we think of the astonishing results of enlightened modern prophylactics as applied by the Japanese medical department of her army.

The method of trying to educate school children by lectures, tracts, and popular treatises on physiology and hygiene, as adopted by some European cities in primary schools under government control, has proved and will continue to prove futile. It is not in accordance with modern ideas of education. To the increasing requirements of a heavy curriculum, the addition of lectures on sanitation is like pumping wind into an already over-inflated toy balloon. We can have nothing to expect but the bursting of the balloon and the loss of all our carefully devised labor. Or, as a friend suggests, like the pumping of air into a punctured tire.

By the plan proposed in this paper the simple practical ocular demonstration will so fully illustrate the principle, that children and adults will absorb a knowledge of hygiene which cannot be grasped as a concept by any other plan, and oral prophylaxis will rapidly become a twentieth century necessity.

